# Mechanistic insights into the therapeutic effects on liver fibrosis in Wilson's disease: a transcriptomic and network pharmacology-based approach

**DOI:** 10.3389/fmed.2025.1581623

**Published:** 2025-06-10

**Authors:** Ying Ma, Yue Pu, Hong Chen, Lei Zhou, Bo Yang, Xiaofeng Huang, Juan Zhang

**Affiliations:** Department of Neurology, The First Clinical Medical College of Anhui University of Chinese Medicine, Hefei, China

**Keywords:** Bushen Huoxue Huazhuo Formula, Wilson's disease, liver fibrosis, LncRNA, network pharmacology

## Abstract

**Background:**

The mechanism of the Bushen Huoxue Huazhuo Formula (BSHXHZF) in treating Wilson's disease (WD) liver fibrosis was investigated in this study using transcriptomics, network pharmacology, and molecular docking approaches.

**Methods:**

Differentially expressed long non-coding RNAs (DELncRNAs) and messenger RNAs in the liver tissues of different groups were identified using high-throughput chip sequencing. Furthermore, the target genes of DELncRNAs were identified, followed by GO functional enrichment and KEGG pathway analyses. DELncRNAs were validated using quantitative reverse transcription-polymerase chain reaction. Active compounds of BSHXHZF and their associated pathways relevant to liver fibrosis treatment in WD, with initial validation via molecular docking.

**Results:**

Transcriptomic analysis identified 63 DELncRNAs by comparing the control with the model and treatment groups. Key DELncRNAs included NONMMUT060008.2, NONMMUT096375.1, and ENSMUST00000153523. Target genes such as *Pik3cd, Pld1, Oprd1, Ppp2r2b*, and *Cers5* were implicated in sphingolipid signaling, metabolism, and AMPK pathways. The “BSHXHZF–Component–Target” network highlighted active ingredients, including tanshinone IIA, quercetin, and luteolin, which play key roles in treating liver fibrosis. Main signaling pathways included IL-17, HIF-1, prolactin, and NF-κB.

**Conclusion:**

The therapeutic effects of BSHXHZF in liver fibrosis associated with WD are likely linked to its modulation of sphingolipid and IL-17 signaling pathways.

## Introduction

Hepatolenticular degeneration, or Wilson's disease (WD), is an autosomal recessive ailment resulting from mutations in *ATP7B*, which is essential for regulating copper metabolism in the body (1). In healthy humans, copper is absorbed from food sources and transported to the liver, where it is integrated into ceruloplasmin, a protein that facilitates copper transport in the bloodstream and is subsequently eliminated into bile. In WD, the faulty ATP7B protein hinders copper transport and excretion, resulting in the excessive accumulation of the metal in the liver and other tissues (2). Although patients with WD may present clinical signs indicative of damage to several organ systems, liver disease is the most common consequence. Excess copper accumulation causes oxidative stress and inflammation, leading to liver cell injury and apoptosis. This cycle of damage and inflammation ultimately results in fibrosis, which affects the normal liver architecture and functionality as it advances, culminating in cirrhosis (3). Furthermore, copper accumulates in the brain, especially in areas such as the basal ganglia, causing neurological and psychiatric manifestations. This phenomenon highlights the varied clinical presentations of the disease, encompassing hepatic failure, mobility problems, and behavioral alterations. The diagnosis is validated using biochemical assays, genetic analyses, and imaging studies. Treatment alternatives comprise the administration of chelating agents to eliminate surplus copper from the body and zinc supplements to block copper absorption (4). Liver failure can aggravate the neurological and psychiatric symptoms associated with WD, emphasizing the critical relationship between hepatic and neurological health. Therefore, promptly identifying and treating liver involvement in WD is crucial to avert irreparable damage and enhance patient prognosis.

This study examined the modulation of long noncoding RNA (LncRNA) expression by the Bushen Huoxue Huazhuo Formula (BSHXHZF) and its effect on liver fibrosis progression. The formulation contains *Rheum palmatum* L. (Dahuang, DH), *Lysimachia christinae* Hance (Jinqiancao, JQC), *Alisma plantago-aquatica* Linn. (Zexie, ZX), *Coptis chinensis* Franch. (Huanglian, HL), *Salvia miltiorrhiza* Bunge (Danshen, DS), *Curcuma phaeocaulis* Valeton (Ezhu, EZ), *Curcuma longa* L. (Jianghuang, JH), *Rehmannia glutinosa* (Gaertn.), DC. (Shudihuang, SDH), *Schisandra chinensis* (Turcz.), Baill. (Wuweizi, WWZ), and *Morinda officinalis* F.C. (Bajitian, BJT). All plant names were verified using “The Plant List” (http://www.theplantlist.org). Previous studies by our research group have observed that BSHXHZF, in combination with bone marrow mesenchymal stem cells, possibly mitigates liver fibrosis and hepatocyte death by alleviating the associated metabolic disturbances, offering therapeutic benefits in WD liver fibrosis. In addition, the active ingredients in BSHXHZF have been identified using liquid chromatography–mass spectrometry (5).

In WD, copper accumulation leads to oxidative stress and hepatocyte death. The damaged hepatocytes release proinflammatory factors and signaling molecules, activating surrounding immune cells such as macrophages and lymphocytes. These activated immune cells produce several inflammatory factors, including tumor necrosis factor-α (TNF-α) and interleukins (IL-1β and IL-6), which further promote the activation of hepatic stellate cells (HSCs) (6, 7). Moreover, the inflammatory response results in the generation of excessive reactive oxygen species (ROS), which not only directly damage the hepatocytes but also activate the immune cells and fibrosis-related signaling pathways, exacerbating HSC activation, a critical step in liver fibrosis (8). Stimulated by liver injury and inflammation, quiescent HSCs transition into an activated state, exhibiting myofibroblast characteristics and secreting large amounts of extracellular matrix (ECM) components, particularly collagen, leading to fibrosis and structural alterations in the liver tissue. In summary, a complex interplay among copper accumulation, oxidative stress, immune cell activation, and HSC activation is responsible for the progression of liver fibrosis. The condition can be reversed when no underlying causes are present (9). Therefore, inhibiting HSC activation is a potential therapeutic target for liver fibrosis. LncRNAs are a class of RNA molecules longer than 200 nucleotides that do not encode proteins but play regulatory roles in several biological processes (BPs), including HSC activation and fibrosis. Various LncRNAs have been identified as key regulators in the activation of HSCs. For instance, LncMEG3 may inhibit the activation of HSCs by targeting NLRC5, facilitating the reversal of liver fibrosis (10). LncRNA GAS5 acts as a microRNA sponge, competitively decreasing its expression levels and effectively inhibiting liver fibrosis (11). Lnc-MALAT1 in the fibrotic liver triggers the activation of HSCs, which in turn augments their fibrogenic activity (12). Silencing Lnc-Lfar1 regulates macrophage pyroptosis, a novel type of proinflammatory programmed cell death, to combat liver fibrosis (13). These findings suggest that abnormal expressions or mutations of LncRNA are closely linked to the occurrence and progression of liver fibrosis and serve as new biomarkers and therapeutic targets for treating the condition.

In this study, a synergistic network pharmacology and transcriptomics approach was employed to identify the key components, targets, and pathways of BSHXHZF for treating WD liver fibrosis. Molecular docking techniques and quantitative reverse transcription-polymerase chain reaction (qRT-PCR) analysis were utilized for the preliminary validation of the findings. The results provided robust evidence for the effective management of WD liver fibrosis.

## Materials and methods

### Animals and experimental design

Four-month-old toxic milk (TX) mice were procured from the Jackson Experimental Center and housed in a specific pathogen-free level animal facility. The TX mice, derived from the drug-likeness (DL) strain, carry a natural genetic defect caused by a spontaneous recessive point mutation at position 2135 in exon 8 of *ATP7B* (14). These mice exhibit pathological processes and copper biochemical characteristics similar to those of patients with WD, making them the most stable and ideal animal model currently available for WD. All animal protocols were approved by the Institutional Animal Care and Use Committee of Anhui Agriculture University (permit number AHAU2023043).

**Figure 1:**
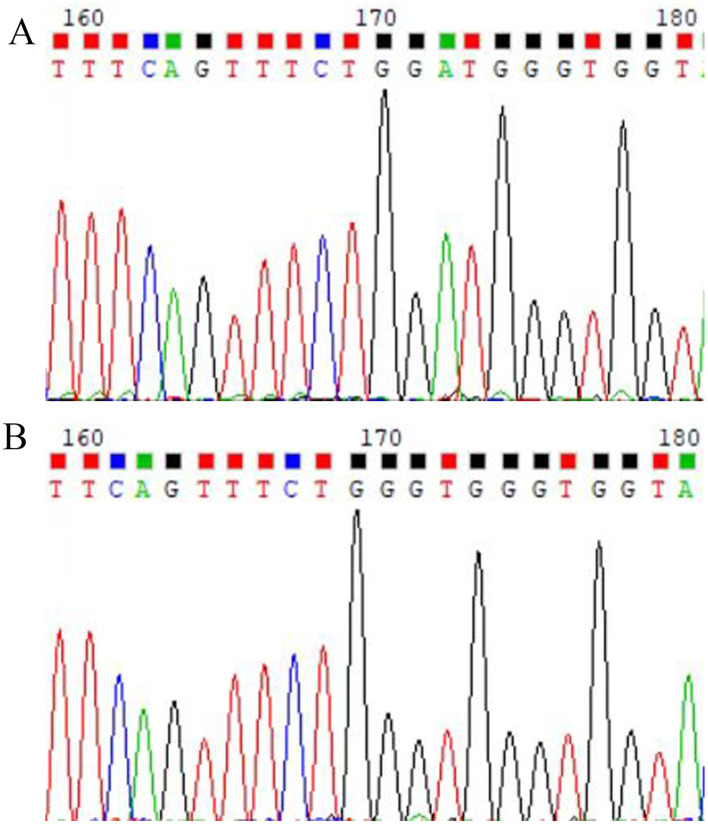
Sequencing map of TX mice. **(A)** Homozygous type; **(B)** wild type.

The environment, maintained at 22°C−26°C with approximately 55% relative humidity, normal lighting conditions, standard rodent feed, and *ad libitum* access to water, was set for the experiment, which began 1 week after acclimation. Twenty TX mice were randomly assigned to two groups, namely the model (NL) and traditional Chinese medicine (TCM) treatment (T) groups. The control group (N) comprised 10 DL mice. Both TX and DL mice were confirmed via gene testing (see Figure 1 for results).

T group: BSHXHZF contains *Rheum palmatum* L. (Dahuang, DH), *Lysimachia christinae* Hance (Jinqiancao, JQC), *Alisma plantago-aquatica* Linn. (Zexie, ZX), *Coptis chinensis* Franch. (Huanglian, HL), *Salvia miltiorrhiza* Bunge (Danshen, DS), *Curcuma phaeocaulis* Valeton (Ezhu, EZ), *Curcuma longa* L. (Jianghuang, JH), *Rehmannia glutinosa* (Gaertn.) DC. (Shudihuang, SDH), *Schisandra chinensis* (Turcz.), Baill. (Wuweizi, WWZ), and *Morinda officinalis* F.C. (Bajitian, BJT). All plant names were verified using “The Plant List” (http://www.theplantlist.org). The concentration of T group was 6.90 g-kg^−1^-d^−1^ (5). The N and NL groups were gavaged an equal volume of physiological saline.

After feeding each group for 4 weeks, the mice were fasted without water for 12 h, anesthetized with sodium pentobarbital (2 ml/kg) via intraperitoneal injection, and the liver was excised. A portion of the organ was fixed in 4% (v/v) paraformaldehyde, dehydrated, cleared with xylene, embedded in paraffin, and serially sectioned for pathological analysis. Another section was placed in a package, sealed in a freezing tube, and stored at −80°C.

### RNA sequencing (RNA-seq)

In brief, the total RNA was directly extracted using the RNeasy Mini Kit (QIAGEN, Germany). RNA integrity was evaluated using an Agilent 5300 with an RNA integrity number (RIN) of >7. For RNA-Seq library construction, ribosomal RNA was removed from DNase I-treated RNA according to the benchmark TruSeq protocol. After preparing the libraries, they were sequenced with the Illumina HiSeq 2500 system.

### Lncrna and messenger RNA (mRNA) sequencing data analysis

FastQC was used to assess the quality of the raw reads, which were subsequently processed to eliminate those with adapters and those of low quality. The reads were mapped using TopHat, allowing for two mismatches. The expression levels of genes in the liver were represented in FPKM (fragments per kilobase of transcript per million mapped reads) and calculated using the TopHat and Cufflinks software packages (15). The RNA-seq data were grouped by selecting transcripts with class codes “i,” “r,” “u,” “x,” and “.” as novel long transcripts. The CPAT (16) software was used for prediction to identify credible LncRNAs. Furthermore, a training dataset of 10,000 mRNA sequences and an equal number of randomly selected subsequences was used to evaluate a mouse-specific cutoff CPAT score via area under the curve analysis of Ensembl coding genes. A cutoff value of 0.487 was selected owing to its optimal sensitivity and specificity, with any sequences not documented in BLASTX considered potential new LncRNAs. Finally, the LncRNAs were identified, quantified, and classified based on their positional relationship with mRNAs.

### Co-expression network analysis

To investigate the interactions between LncRNAs and mRNAs, a gene coexpression network was constructed based on the normalized FPKM values of individual genes. After screening for differentially expressed LncRNAs (DELncRNAs) and differentially expressed mRNAs (DEmRNAs), the Pearson correlation coefficients (PCCs) between them were calculated. Significant LncRNA–mRNA pairs with correlations defined as |PCC| > 0.98 and *P* < 0.05 were retained. This analysis permitted the identification of high-quality correlations between the DELncRNAs and DEmRNAs. Enrichment analysis was performed using the GOseqR package to explore the potential roles of the target genes regulated by LncRNAs. Furthermore, Gene Ontology (GO) analysis was conducted on the differentially expressed protein-coding genes. Subsequently, significantly enriched GO and Kyoto Encyclopedia of Genes and Genomes (KEGG) pathways were identified using a corrected *P* of < 0.05.

### Network pharmacology analysis

A search was performed using the TCMSP database (https://old.tcmsp-e.com/tcmsp.php) with the keywords DH, JQC, ZX, HL, DS, EZ, JH, SDH, WWZ, and BJT from BSHZXHZF. During the screening process, the criteria were set as ≥30% for oral bioavailability and ≥0.18 for DL to identify the possible active components in each group and their corresponding potential targets. The component targets were then converted to standard gene names using the UniProt database (http://www.uniprot.org/), selecting the species “*Homo sapiens*” to obtain relevant target information.

Input the screened active components and targets into Cytoscape software to construct a “Traditional Chinese Medicine—Active Component—Target” network diagram, and select important targets based on degrees greater than twice the median value.

Using the keywords “Wilson's disease” and “liver fibrosis,” a search was performed for disease targets related to WD via the GeneCards (https://www.genecards.org), OMIM (https://omim.org), and DrugBank (https://go.drugbank.com) databases. The targets in each database were recorded and merged to eliminate duplicates.

The targets of BSHXHZF were intersected with those of WD liver fibrosis, and a Venn diagram was created. The data were uploaded to the STRING 11.0 database with a high confidence interaction threshold (0.700), and discrete nodes were hidden to obtain the protein–protein interaction (PPI) network. The relevant information was imported into the Cytoscape software to filter for core targets. Metascape (https://metascape.org) was used to perform GO functional analysis and KEGG pathway analysis on the common targets of BSHXHZF and WD. The Microbioinformatics website (https://www.bioinformatics.com.cn) was utilized for visualization.

### Molecular docking

The three-dimensional structures of key target proteins were obtained from the PubChem database (https://pubchem.ncbi.nlm.nih.gov/). Operations such as removing crystallization water and adding hydrogen atoms to the proteins were performed using AutoDockTools 1.5.6. Molecular docking and visualization of the docking results were then conducted using AutoDock Vina 1.1.2, along with PyMOL 2.1 and Discovery Studio V2019 (Dassault Systèmes Biovia, San Diego, CA, USA). The results were assessed based on binding free energy, with lower energy values indicating a more stable conformation of the compound when binding to the receptor and, in turn, a higher likelihood of interaction and more reliable docking outcomes.

### Quantitative RT-PCR

The RNA was subsequently transcribed into cDNA using the RevertAid First Strand cDNA Synthesis Kit (#K1622, Thermo, USA) for LncRNA and gene analysis. The LncRNA and gene levels were determined using the HieffTM qPCR SYBR^®^ Green Master Mix (No Rox Plus) 11201ES (11201ES03, Shanghai Yeasen BioTechnologies, China). The reaction conditions were as follows: 95°C for 30 s, followed by 40 cycles at 95°C for 15 s and 60°C for 30 s. All gene expressions were calculated using the 2^−ΔΔCt^ method and normalized to GAPDH. The primers were synthesized by Tian Yi Hui Yuan Biotechnology Co., Ltd. (Beijing, China). The primer sequences are shown in Supplementary Table 1.

### Statistical analysis

In transcriptomic analysis, FastQC was first used for data quality control to remove low-quality reads and adapter sequences. HISAT2 (http://ccb.jhu.edu/software/hisat2/index.shtml) was employed for sequence alignment, followed by transcript assembly using Cufflinks (http://cole-trapnell-lab.github.io/cufflinks/). CPAT combined with BLASTX was used to screen non-coding RNA, and DESeq2 was applied for differential expression analysis, with results visualized using volcano plots. Data normality was assessed using the Shapiro–Wilk test. Normally distributed data are presented as the mean ± standard deviation and analyzed by one-way analysis of variance, followed by least-significant difference or Welch's test for between-group comparisons. Non-normally distributed data were transformed or analyzed using non-parametric tests (e.g., Kruskal–Wallis test). All statistical analyses were performed using SPSS v26.0 (IBM, USA), with a significance threshold of *P* < 0.05.

## Results

### BSHZHZF-induced alleviation of liver fibrosis

**Figure 2:**
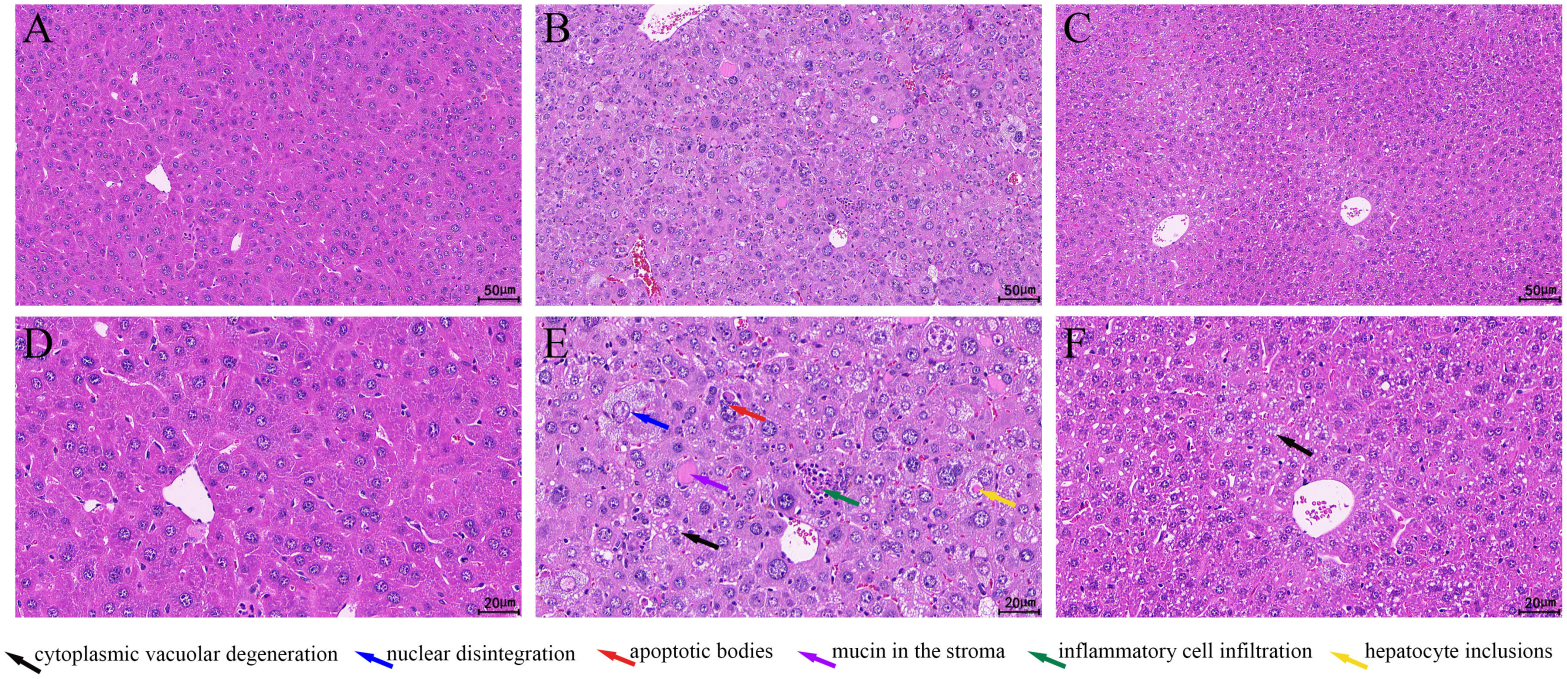
H&E staining of the liver. **(A, D)** N group; **(B, E)** NL group; **(C, F)** T group. A.B.C:Bar = 50 μm; D.E.F:Bar = 20 μm.

The liver tissue of the N group appeared normal (Figures 2A, D), with clear hepatocyte outlines, dense cytoplasm, regular morphology, and no significant degeneration. The liver cords were arranged in an orderly manner, without any apparent infiltration of inflammatory cells. In contrast, the liver tissue of the NL group showed severe abnormalities (Figures 2B, E), with widespread hepatocyte degeneration, cellular swelling, and cytoplasmic vacuolar degeneration (Figure 2E, black arrow). Certain hepatocytes exhibited necrosis, with obvious nuclear degeneration (Figure 2E, blue arrow). Apoptotic bodies (Figure 2E, red arrow) and hepatocyte inclusions (Figure 2E, yellow arrow) were visible. Inflammatory cell infiltration was noted within the lobules (Figure 2E, green arrow), and mucin was present in the stroma (Figure 2E, purple arrow). The T-group displayed significant improvement in liver damage when compared to the model group (Figures 2C, F), with only mild edema in some hepatocytes and cytoplasmic vacuolar degeneration (Figure 2F, black arrow). The nuclei appeared relatively normal without any swelling, and no significant infiltration of inflammatory cells was observed in the tissue.

### BSHXHZF attenuates WD liver fibrosis via three key DELncRNAs

**Figure 3:**
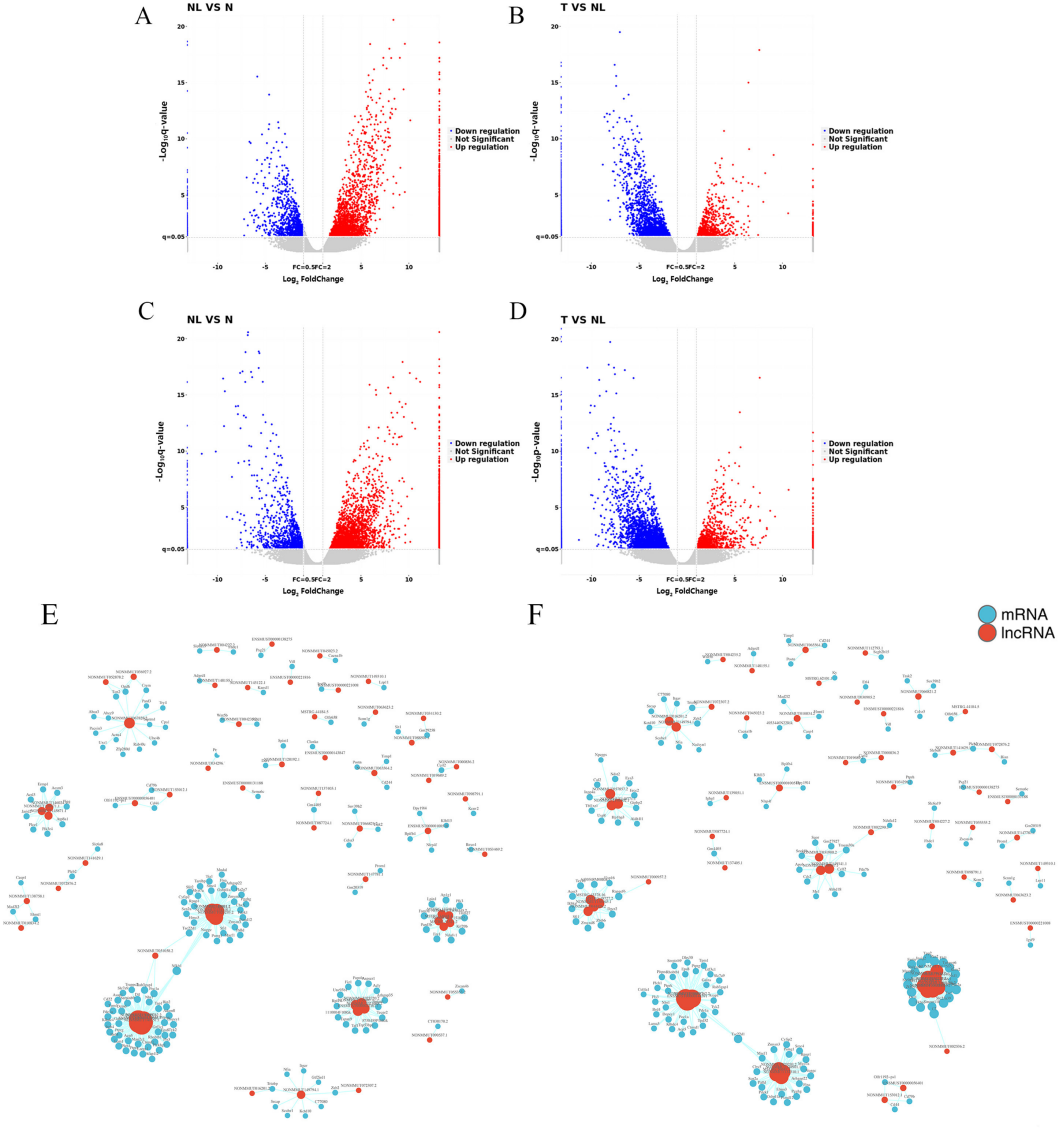
The discrete expression patterns of mRNAs and lncRNAs. Volcano plot for lncRNAs **(A, B)** and mRNAs **(C, D)**. **(A, C)**: NL vs. N, **(B, D)**: T vs. NL. Signal pathway networks of lncRNAs involved in DElncRNA-mRNA relationships. Co-expression of DElncRNA-mRNA after lncRNA targeting coincided with DE mRNAs in **(E)** NL vs. N and **(F)** T vs. NL.

The LncRNA expression profile was examined by performing deep RNA-Seq experiments on mouse liver tissues from the N, NL, and T (*n* = 4 per group) groups. A total of 63,840 known and 1064 novel LncRNAs were identified in the liver samples. To elucidate the roles of LncRNAs in various models, RNA-Seq was performed to obtain their expression profiles. The comparison of the NL group with the N group and the T group with the NL group revealed 2,690 (1,798 upregulated and 892 downregulated) and 2,133 (616 upregulated and 1,517 downregulated) DELncRNAs, respectively (Figures 3A, B). In addition, 3,379 and 3,061 DEmRNAs, respectively, were identified; of these, 2,399 were upregulated and 980 were downregulated in the first comparison, and 867 were upregulated and 2,194 were downregulated in the second (Figures 3C, D). The top 10 most significant DELncRNAs are listed in Tables 1, 2. DELncRNAs with significant biological relevance were identified, including NONMMUT060008.2, NONMMUT096375.1, and ENSMUST00000153523, which are likely to play key roles in WD liver fibrosis.

**Table 1:** The top 10 DElncRNAs between the NL and N groups.

**lncRNA_ID**	**Locus**	**Length**	**NL**	**N**	**log_2_FC**	***P*-value**	**UP/DOWN**
NONMMUT060008.2	7:14410690–14411717	1,028	0.172	172.514	−9.973	2.52E-41	DOWN
NONMMUT060075.2	7:16915382–16916322	941	0.067	27.846	−8.690	2.63E-38	DOWN
NONMMUT149510.1	5:52972972–52992405	17,201	0.001	0.101	−7.231	2.76E-05	DOWN
NONMMUT021638.2	14:75176865–75182956	6,068	0.002	0.285	−7.224	0.00206	DOWN
NONMMUT036430.2	2:27540440–27546088	3,486	0.014	1.795	−6.964	1.49E-09	DOWN
NONMMUT096375.1	15:89767563–89768777	817	0.057	6.597	−6.844	0.00013	DOWN
ENSMUST00000153523	2:173124749–173133204	3,476	0.014	1.462	−6.672	4.11E-13	DOWN
NONMMUT147062.1	2:147944002–148040242	1,971	0.006	0.572	−6.605	5.29E-06	DOWN
NONMMUT152860.1	8:105051546–105058411	1,236	0.037	3.502	−6.546	5.83E-13	DOWN
NONMMUT008761.2	11:20225237–20226853	1,617	0.206	17.552	−6.414	1.12E-05	DOWN
MSTRG.61463.4	9:58702406–58712258	1,161	28.375	0.017	10.718	2.71E-31	UP
NONMMUT089188.1	13:3363013–3363773	761	14.982	0.012	10.239	1.44E-29	UP
NONMMUT050450.2	4:144131848–144133881	2,034	59.925	0.053	10.152	3.13E-15	UP
MSTRG.47803.6	5:145463518–145800965	1,195	12.340	0.016	9.584	1.03E-22	UP
NONMMUT139051.1	1:71891107–71897667	4,939	1.516	0.002	9.581	4.49E-08	UP
MSTRG.40589.1	4:40651444–40660004	297	22.185	0.032	9.448	2.50E-18	UP
MSTRG.64312.1	X:33541135–33841298	643	20.056	0.030	9.365	1.20E-25	UP
NONMMUT089191.1	13:3386160–3388230	2,071	5.325	0.009	9.139	3.52E-27	UP
MSTRG.36679.1	3:12833226–12849812	1,630	3.330	0.006	9.117	1.60E-10	UP
MSTRG.64316.1	X:33549148–33863315	1,414	3.692	0.007	9.060	2.41E-21	UP

**Table 2:** The top 10 DElncRNAs between the T and NL groups.

**lncRNA_ID**	**Locus**	**Length**	**T**	**NL**	**Log_2_FC**	***P*-value**	**UP/DOWN**
MSTRG.62401.1	9:89987080–90046512	3,427	0.004	1.673	−8.799	4.24E-11	DOWN
MSTRG.66097.1	Y:39883592–39893879	2,204	0.018	7.174	−8.623	5.84E-16	DOWN
NONMMUT118974.1	5:104600696–104602539	1,844	0.007	2.305	−8.368	2.64E-16	DOWN
NONMMUT105034.1	18:86385669–86386863	1,195	0.011	3.395	−8.301	1.64E-15	DOWN
NONMMUT025368.2	16:7870102–7879859	3,886	0.003	0.979	−8.208	2.24E-07	DOWN
NONMMUT072441.2	X:52553142–52556050	2,909	0.004	1.227	−8.116	1.39E-14	DOWN
NONMMUT074758.2	Y:1153078–1157029	3,952	0.003	0.880	−8.078	3.20E-16	DOWN
ENSMUST00000221818	12:111950517–111954152	3,556	0.004	0.949	−8.035	2.51E-05	DOWN
MSTRG.64934.1	X:86533000–86536296	1,053	0.011	2.364	−7.757	2.05E-10	DOWN
ENSMUST00000200644	5:70561265–70564103	2,839	0.014	2.836	−7.705	5.96E-15	DOWN
NONMMUT142366.1	13:52180149–52191792	10,785	5.461	0.004	10.601	5.05E-06	UP
NONMMUT137216.1	X:103460376–103483218	17,377	0.679	0.001	9.791	2.10E-28	UP
ENSMUST00000127786	X:103460506–103483217	17,769	36.570	0.068	9.076	4.05E-12	UP
NONMMUT060008.2	7:14410690–14411717	1,028	50.312	0.172	8.195	3.20E-10	UP
NONMMUT152503.1	8:10869829–10892063	6,416	0.531	0.002	7.999	3.04E-07	UP
ENSMUST00000153523	2:173124749–173133204	3,476	2.740	0.014	7.579	8.31E-23	UP
NONMMUT096375.1	15:89767563–89768777	817	10.352	0.057	7.494	7.95E-09	UP
NONMMUT151597.1	7:39923037–39938708	3,607	0.634	0.004	7.455	0.001534	UP
NONMMUT146565.1	2:166194880–166203583	7,625	0.348	0.003	6.839	4.23E-09	UP
NONMMUT034722.2	19:41210852–41213976	3,125	4.524	0.048	6.555	3.66E-05	UP

### DELncRNAs-mrnas co-expression network regulatory relationships in WD liver fibrosis

To better understand the relationship between LncRNAs and mRNAs, a coexpression network of DELncRNAs and differentially expressed target mRNAs was constructed, representing the regulatory roles of LncRNAs and mRNAs in WD liver fibrosis. In the network between the NL and N groups, 63 LncRNAs and 155 mRNAs were identified (Figure 3E). Moreover, 63 LncRNAs and 150 mRNAs were noted between the T and NL groups (Figure 3F). Most coexpressions between these LncRNAs and mRNAs were positively correlated, showing the involvement of these regulatory relationships in WD liver fibrosis. In addition, one LncRNA was coexpressed with several mRNAs, while multiple LncRNAs were associated with a single mRNA. These findings highlight the presence of an intricate regulatory relationship within the differential co-expression network.

### Common target genes in the sphingolipid pathway underlie BSHXHZF's regulatory effects on WD liver fibrosis

**Figure 4:**
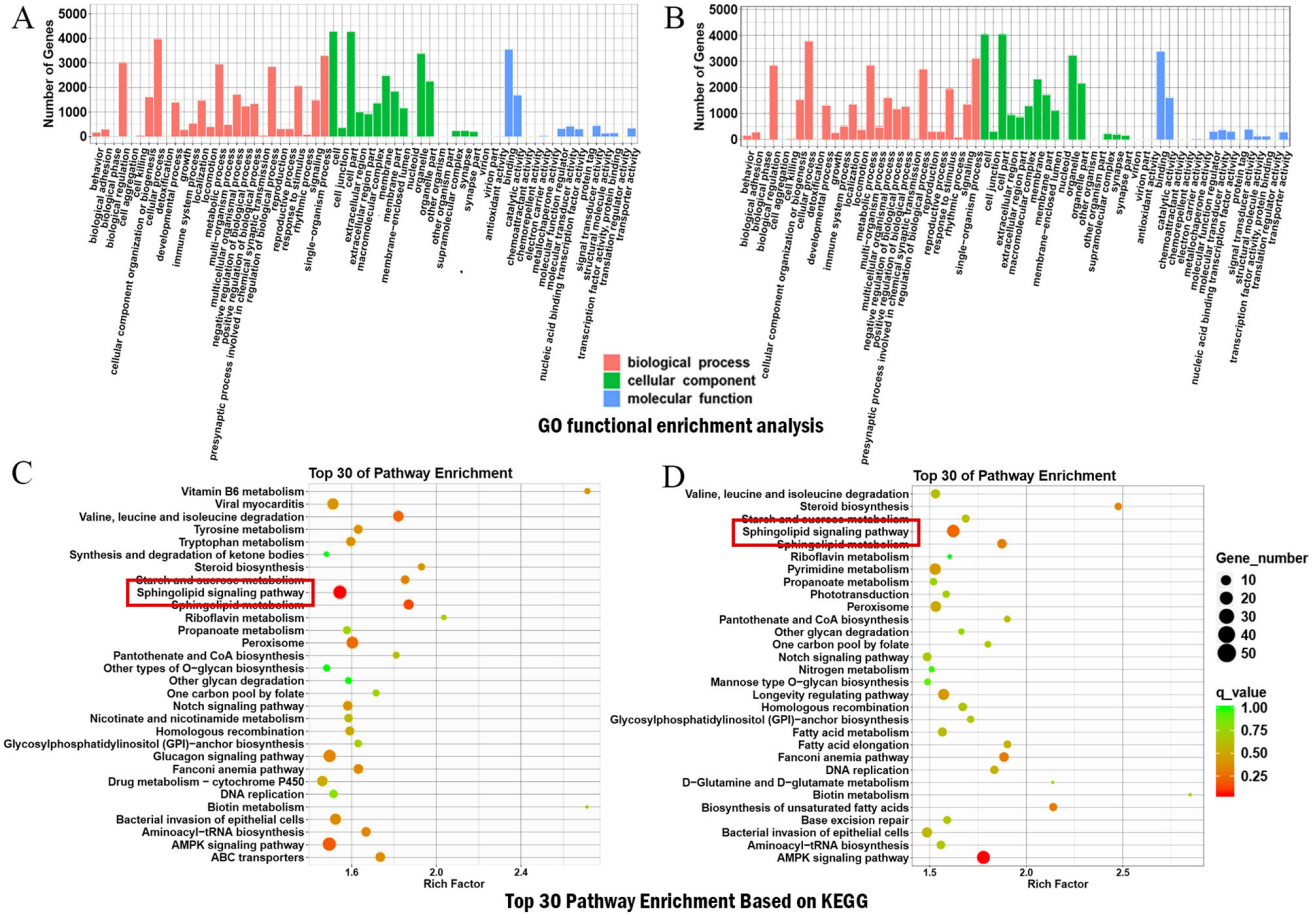
Enrichment analysis of target genes. **(A, B)** GO function analysis of the DElncRNAs target genes in NL vs. N, and T vs. NL. **(C, D)** KEGG enrichment analysis of DElncRNAs target genes in NL vs. N, and T vs. NL.

The target genes predicted from the DELncRNAs, derived from pairwise comparisons of each group, were subjected to GO and KEGG functional annotation and enrichment analyses. In the NL vs. N group and the T vs. NL group, GO functional enrichment analysis revealed that the target genes linked to DELncRNAs were prominently categorized under BPs as cellular process, single-organism process, and biological regulation. In cellular components (CCs), they were classified as cells and cell parts, and in molecular functions (MFs), binding was particularly significant (Figures 4A, B). The protein-coding genes were further subjected to KEGG (Figures 4C, D) functional enrichment analyses, and the top 30 terms were identified. In the comparison of the NL vs. N group, the target genes were primarily enriched in the sphingolipid signaling pathway, sphingolipid metabolism, and AMPK signaling pathway. Finally, in the T vs. NL group comparison, the target genes were significantly enriched in the AMPK signaling pathway, sphingolipid signaling pathway, and metabolic pathways. Hence, BSHXHZF may alleviate liver fibrosis damage in WD mice by affecting the sphingolipid signaling pathway. The common target genes between the sphingolipid signaling pathway and the DELncRNA target genes were selected, with 16 common target genes in the NL vs. N group and 14 in the T vs. NL group. Of these, 12 genes—*Pik3cd, Pld1, Oprd1, Gab2, Abcc1, Ppp2r2b, Cers5, Bcl2, Rac2, Adora3, Sgpp2*, and *Sphk1*—were upregulated in the NL vs. N group and downregulated in the T vs. NL group (Table 3). BSHXHZF exerted a significant inhibitory effect on the 12 upregulated genes.

**Table 3:** The folds of 12 common target genes.

**Genes name**	**Log_2_FC (NL vs. N)**	**Log_2_FC (T vs. NL)**
Sphk1	2.444	−2.567
Sgpp2	4.212	−3.143
Rac2	1.934	−1.633
Bcl2	1.732	−1.747
Cers5	1.612	−1.658
Ppp2r2b	4.063	−4.655
Abcc1	1.971	−2.019
Oprd1	6.564	−3.546
Gab2	2.466	−2.080
Pik3cd	1.873	−1.596
Pld1	1.847	−1.897
Adora3	2.764	−3.408

### BSHXHZF ameliorates WD liver fibrosis via multi-component synergy targeting 15 key genes

**Figure 5:**
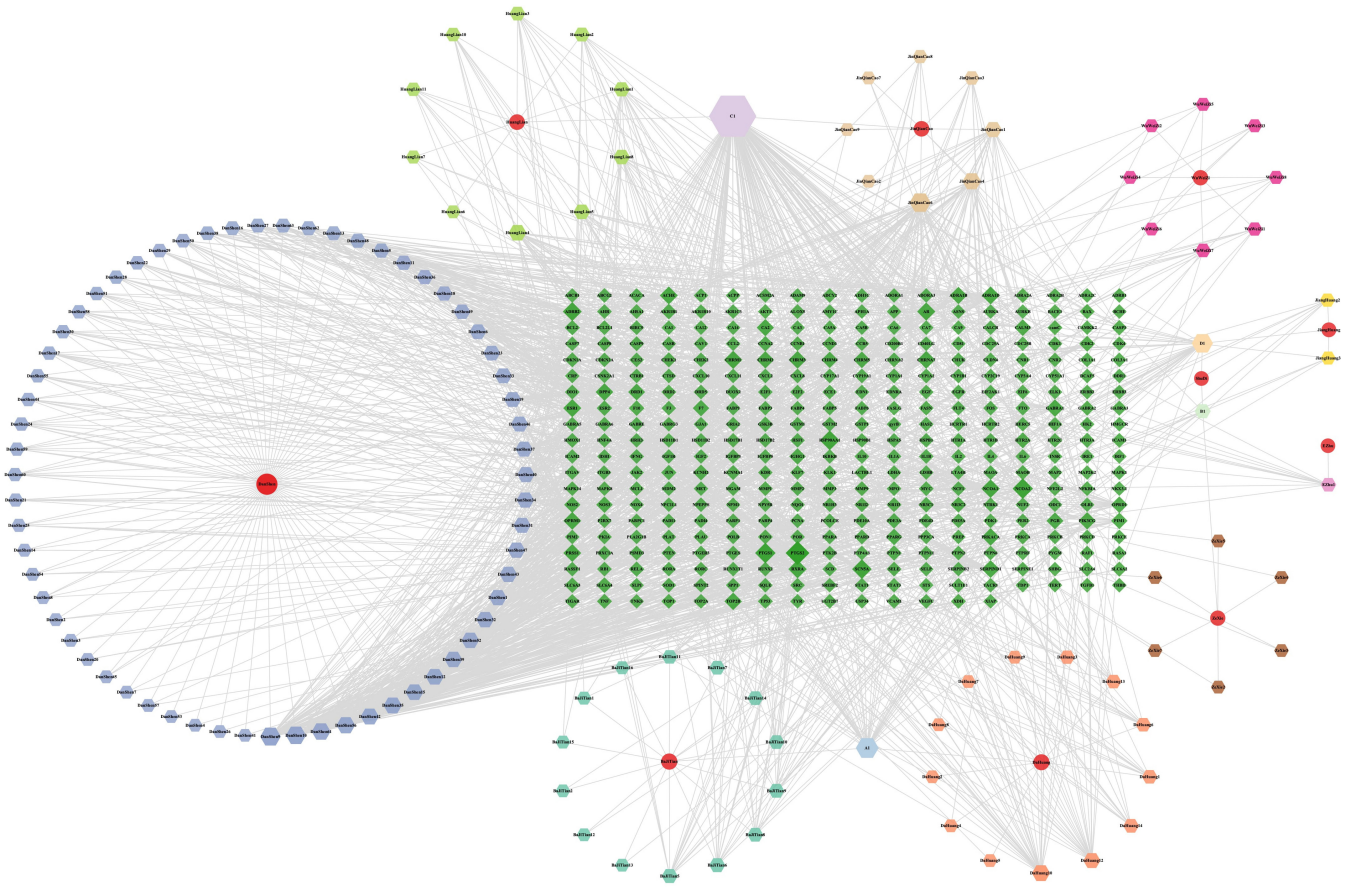
The “BSHXHZF-ingredient-target” network. The “BSHXHZF-ingredient-target” network. The circles, hexagons, and diamonds correspond to the herb, ingredient, and target, respectively.

**Figure 6:**
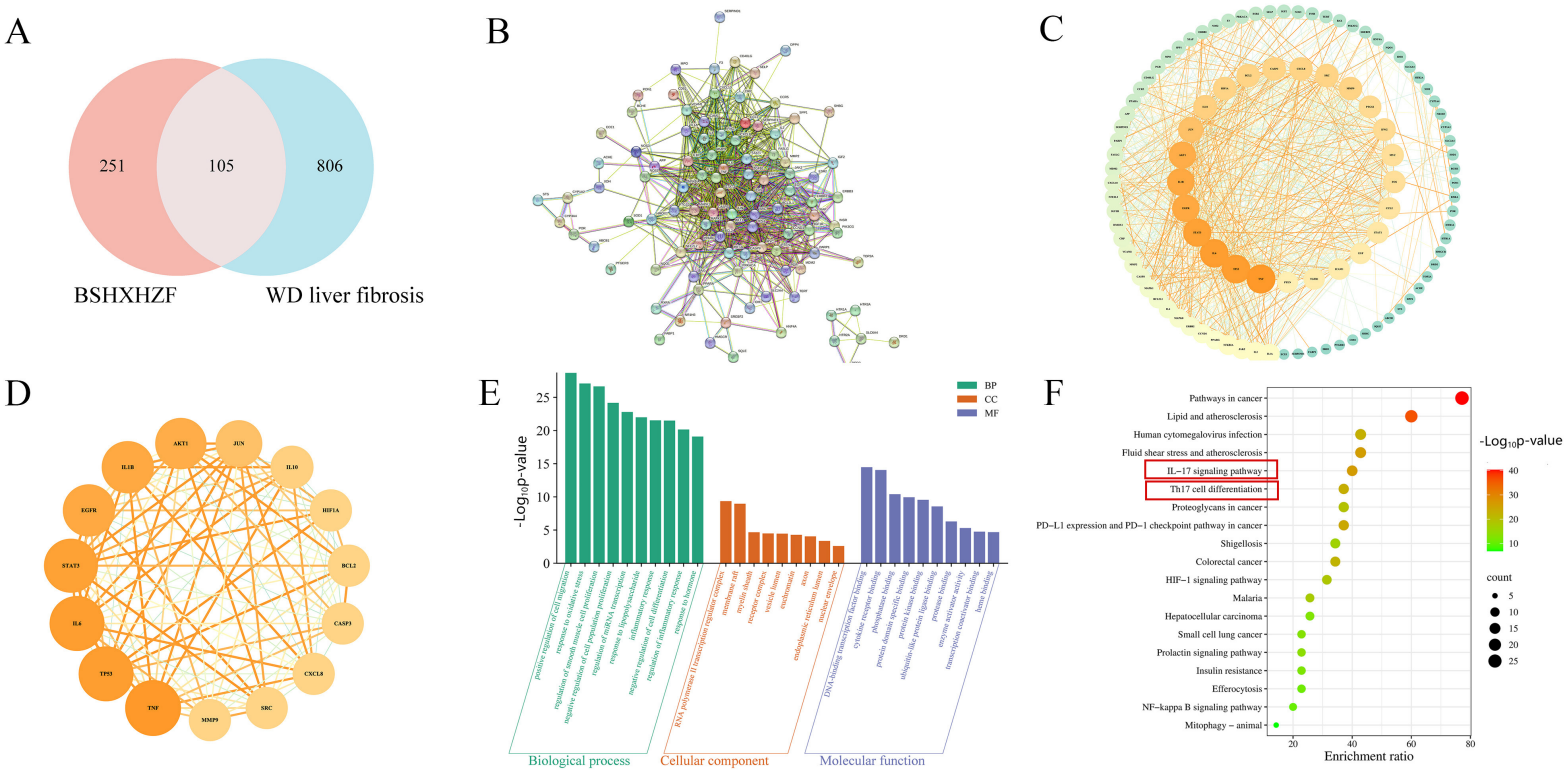
Screening of HUB genes. **(A)** Venn diagram of BSHXHZF (pink) and WD liver fibrosis genes (blue). **(B)** The PPI network was derived from the STRING database, using a minimum required interaction score of 0.90. **(C)** Visualization of the PPI network of 101 target genes. **(D)** Map of the top 15 core target genes from the PPI network. The node sizes range from small to large, with colors transitioning from yellow to dark red, reflecting a gradual increase in interactions. GO and KEGG enrichment analysis. **(E)** The GO analysis identifies 10 relevant pathways from each of the three processes. **(F)** Top 19 KEGG pathways from screening.

A total of 134 active components were identified, leading to 129 unique bioactive components after eliminating the duplicates. The targets of these components in BSHXHZF were predicted, resulting in a final count of 356 genes after further removing the duplicate targets. Based on the above data, the “BSHXHZF–ingredient–target” network was constructed using Cytoscape, which comprised 495 nodes and 1,938 edges (Figure 5). Moreover, the following five key ingredients were identified using the method mentioned above: tanshinone IIA (MOL007154), quercetin (MOL000098), luteolin (MOL000006), α-amyrin (MOL006824), and kaempferol (MOL000422). In addition, 911 targets related to WD liver fibrosis were screened, and 105 intersecting targets were identified between BSHXHZF and WD liver fibrosis. PPI network analysis of the 101 intersecting targets (four of which did not exhibit a discrete distribution) was performed using the STRING database (Figure 6A). As depicted in Figure 6B, the network comprised 101 nodes and 902 edges, with an average node degree of 17.9. The PPI network of 101 target genes was visualized (Figure 6C). By constructing the PPI network, the associations between these target genes can be visually presented. The topological features of the “network analysis” tool in Cytoscape were evaluated using “DC,” “BC,” “CC,” and “stress.” The median values were utilized as the criteria, and the 35 HUB genes were identified using DC ≥14, BC ≥0.004, CC ≥0.487, and stress ≥544. Cytoscape aided in visualizing the HUB genes by assigning large sizes and bright colors for low degree values and small sizes and dark colors for high degree values. A total of 15 core targets with DC ≥37 were identified (Figure 6D and Table 4).

**Table 4:** DC, BC, CC, and stress information of 15 core targets.

**No**.	**Name**	**Degree**	**BetweennessCentrality**	**ClosenessCentrality**	**EdgeCount**	**Stress**
1	TNF	52	0.060	0.667	52	5,270
2	TP53	51	0.090	0.648	51	5,660
3	IL6	51	0.049	0.653	51	5,034
4	STAT3	50	0.077	0.648	50	4,942
5	EGFR	48	0.055	0.631	48	4,374
6	IL1B	48	0.044	0.648	48	4,612
7	AKT1	47	0.049	0.618	47	3,834
8	JUN	42	0.027	0.618	42	2,758
9	IL10	38	0.015	0.588	38	2,070
10	BCL2	37	0.019	0.563	37	1,964
11	HIF1A	37	0.018	0.577	37	1,854
12	CXCL8	37	0.014	0.573	37	1,886
13	MMP9	37	0.013	0.580	37	1,832
14	SRC	37	0.021	0.588	37	2,116
15	CASP3	37	0.013	0.577	37	1,350

### BSHXHZF ameliorates WD liver fibrosis primarily via IL-17-signaling pathway and th17 cell differentiation regulation

The Metascape database was used to examine the BPs, CCs, and MFs of the GO terms (Figure 6E) and KEGG pathways (Figure 6F) associated with the 35 HUB genes. In the GO analysis, the key pathways related to BPs suggested that the HUB genes were linked to the positive regulation of cell migration, response to oxidative stress, and regulation of smooth muscle cell proliferation, among others. CCs encompassed pathways associated with the RNA polymerase II transcription regulator complex, membrane rafts, myelin sheaths, and other related structures. MFs were linked to various types of binding, such as DNA, transcription factor, cytokine receptor, and phosphatase binding, among others.

Several KEGG pathways, including IL-17, hypoxia-inducible factor 1 (HIF-1), prolactin, nuclear factor kappa B (NF-κB) signaling pathway, and Th17 cell differentiation, were linked to inflammation, immune regulation, cell survival and proliferation, and metabolic regulation. These pathways are likely to play a critical role in treating WD liver fibrosis using BSHXHZF.

### BSHXHZF reverses WD liver fibrosis by targeting the IL-6/TNF inflammatory axis and modulating key lncrna/gene expression

**Figure 7:**
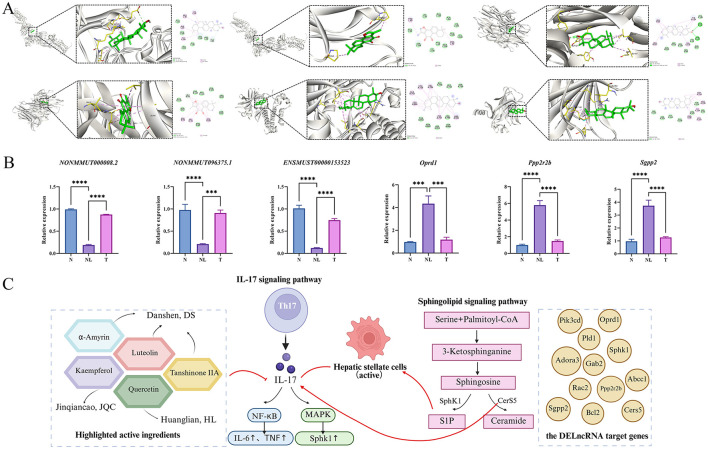
Mechanism validation. **(A)** Molecular docking map of main components of BSHXHZF and core targets. IL6 and the structure for α-Amyrin (top left). IL6 and the structure for Tanshinone (top center). TNF and the structure for α-Amyrin (top right). TNF and the structure for Tanshinone (bottom left). EGFR and the structure for α-Amyrin (bottom center). TP53 and the structure for α-Amyrin (bottom right). **(B)** The three DElncRNAs and target genes extracted from liver tissue were verified by qRT-PCR *n* = 3. Data are presented as mean ± SD, Comparisons between multiple groups were analyzed by one-way ANOVA, followed by Tukey's test for equal variances and Dunnett's T3 test for unequal variances. ***P < 0.001, ****P < 0.0001. **(C)** The anti-fibrotic mechanism of BSHXHZF involves the regulation of sphingolipid and IL-17 signaling pathways.

Molecular docking analysis was performed to evaluate the relationship between the five main components and the core targets: TNF, TP53, IL-6, STAT3, and EGFR. The lower the binding energy, the more stable the docking. A binding energy of < -5.0 kcal·mol^−1^ signifies a strong affinity between the two. The best docking activities (Figure 7A) were perceived in α-amyrin–IL-6 (−7.66), α-amyrin–TNF (−9.24), α-amyrin–TP53 (−7.48), α-amyrin–EGFR (−7.82), tanshinone–IL-6 (−7.61), and tanshinone–TNF (−7.69).

The relative expression levels of the DELncRNAs NONMMUT060008.2, NONMMUT096375.1, ENSMUST00000153523 and the genes *Oprd1, Ppp2r2b*, and *Sgpp2* were determined using qRT-PCR (Figure 7B). The findings indicated that NONMMUT060008.2, NONMMUT096375.1, and ENSMUST00000153523 were significantly downregulated in the WD liver fibrosis model (*P* < 0.0001). Nonetheless, after BSHXHZF treatment, the expression levels of NONMMUT060008.2, ENSMUST00000153523 (*P* < 0.0001), and NONMMUT096375.1 (*P* < 0.001) were significantly upregulated. In contrast, *Ppp2r2b, Sgpp2* (*P* < 0.0001), and *Oprd1* (*P* < 0.001) were significantly upregulated in the WD liver fibrosis model. However, after BSHXHZF treatment, the expression levels of *Ppp2r2b, Sgpp2* (*P* < 0.0001), and *Oprd1* (*P* < 0.001) were significantly downregulated. These results validated our hypothesis that BSHXHZF may play a key role in WD liver fibrosis.

## Discussion

WD is a hereditary metabolic disorder marked by abnormal copper accumulation in the body, severely affecting liver function and resulting in complications such as liver fibrosis, cirrhosis, and acute liver failure (17). As the disease progresses, liver fibrosis becomes a major health concern for the affected patients. The mechanisms underlying this condition are complex and involve the activation of HSCs, deposition of ECM, and regulation of multiple signaling pathways. Hence, examining the association between WD and liver fibrosis is vital for enhancing therapeutic outcomes. Recently, TCM has garnered increasing attention as a treatment approach to combat liver fibrosis (18–20). TCM not only exhibits multitarget effects but also improves the overall state of the body and immune function, ensuring a healthy microenvironment in the liver. For example, certain TCM components, such as DS (21) and JH (22), can inhibit the activation of HSCs, alleviate inflammatory responses, and promote the repair of liver cells. These observations emphasize the unique potential and advantages of TCM in treating liver fibrosis linked to WD, providing novel options for clinical practice. In summary, conducting in-depth investigations on the mechanisms by which TCM addresses liver fibrosis related to WD enriches the theoretical foundation for treatment and offers valuable insights for augmenting patients' quality of life.

This transcriptomic study identified various differentially expressed genes, including *Pik3cd, Pld1, Oprd1, Gab2, Abcc1, Ppp2r2b, Cers5, Bcl2, Rac2, Adora3*, Sgpp2, and *Sphk1*. Changes in the expression of these genes may stimulate the progression of liver fibrosis by affecting cell proliferation, apoptosis, and signaling mechanisms. The PI3K/Akt pathway has emerged as a viable target in antifibrotic therapy owing to its ability to enhance the activation and proliferation of HSCs and increase the production of collagen and other ECM proteins (23, 24). *PIK3CD* is the gene that encodes the PI3K catalytic subunit p110δ; its overexpression elevates the levels of p-Akt, whereas its inhibition exerts the opposite effect (25). PI3K/Akt activation can heighten the levels of the downstream antiapoptotic protein Bcl2, promoting the activation of caspase 3, decreasing the expression of the proapoptotic protein caspase 3, preventing cell apoptosis, and improving liver disease (26). The sphingolipid pathway is a key metabolic pathway within cells that is responsible for the synthesis and degradation of sphingolipid molecules. These complex lipid molecules primarily include ceramide (Cer), sphingosine (Sp), sphingosine-1-phosphate (S1P), and ceramide-1-phosphate (C1P). These molecules play crucial roles in the structure and function of cell membranes, signal transduction, cell proliferation, and apoptosis (27). S1P and its metabolic enzymes are intricately involved in liver fibrosis (28). Furthermore, S1P can not only stimulate the proliferation and migration of HSCs *in vitro* but also trigger the upregulation of ECM proteins (29). *SphK1* phosphorylates sphingosine to S1P, promoting cell proliferation and inhibiting cell apoptosis (30). The SphK/S1P signaling pathway plays a role in the progression of tissue fibrosis. Inhibiting SphK1 expression can effectively reduce liver damage and inflammation (31). *CerS5* is responsible for the synthesis of long-chain ceramides. The plasma levels of ASMase are elevated and ceramide-positive erythrocytes are constitutively increased in WD (32). *CerS5* knockout not only reduces the expression of the key enzyme Cyp27a1 but also elevates the levels of 12a-OH BAs, specifically CAs and DCAs, which play a role in HSC activation and the advancement of liver fibrosis (33). The interactions among these genes suggest that the sphingolipid signaling pathway may become a novel target for treating WD liver fibrosis, providing new therapeutic strategies.

This study used network pharmacology methods to identify potential targets of BSHXHZF for treating WD liver fibrosis, including tanshinone IIA, quercetin, flavonoids, α-amyrin, and hesperidin, as well as signaling pathways, such as IL-17, HIF-1, prolactin, and NF-κB. Tanshinone IIA inhibits the lipopolysaccharide-induced inflammatory response, downregulating the mRNA expression of NF-κB-dependent genes, such as IL-1β, TNF-α, and IL-6, and suppressing HSC activation (34). Tanshinone IIA may regulate liver fibrosis via its antioxidant effects, which could be associated with the PI3K/Akt and Nrf2/HO-1 signaling pathways (35). The antifibrotic effect of quercetin is closely linked to the inhibition of TGF-β1, Smad3, and NF-κB expression, which in turn augments the expression of downstream antioxidant genes and suppresses the release of inflammatory factors (36). Under the combined induction of TGF-β and IL-6, naive CD4^+^ T cells differentiate into Th17 cells secreting IL-6 and IL-17, participating in inflammatory responses and autoimmune diseases. IL-17 can activate HSCs either directly by promoting their proliferation and increasing collagen expression or indirectly by activating other proinflammatory cells (37, 38). The NF-κB signaling pathway can regulate inflammatory cytokines, interfering with the occurrence of liver fibrosis. This specific stimulation may alter the expression of genes associated with immune response, inflammation, and proliferation in the human body, consequently changing the symptoms of liver fibrosis. Molecular docking experiments validated the binding potential between key active components in BSHXHZF and the core targets of inflammation and fibrosis, which has bridged the research gap between transcriptomics/network pharmacology predictions and the potential binding mechanisms. Among these the high binding energies of α-amyrin with TNF and tanshinone IIA with IL-6 indicate that these components can directly inhibit the activity of pro-inflammatory factors and block the abnormal activation of downstream signaling pathways such as IL-17 and sphingolipid metabolism, thereby regulating the LncRNA network associated with HSC activation and, ultimately, alleviating the progression of liver fibrosis.

The results of transcriptomics and network pharmacology analysis demonstrated that sphingolipid and IL-17 signaling pathways play essential roles in treating WD liver fibrosis. Furthermore, the activation of Th-17 lymphocytes is influenced by sphingosine via the activation of the S1P receptor. S1P can enhance the expansion and functional activation of Th17 cells (39). The overexpression of the S1P–S1P1 axis affects the expansion of Th17 subsets, leading to an increase in IL-17 secretion (40). In a mouse model of alcohol-associated steatohepatitis, excessive activation of S1P promoted the differentiation of Th17 cells and the expression of IL-17, exacerbating inflammation and liver damage (41). In the context of liver disease and fibrosis, IL-17 can stimulate the activation of HSCs, while sphingolipid metabolites such as ceramide can enhance the proliferation and activation of HSCs. This interaction may play a key role in promoting fibrosis, suggesting that the synergistic effects of sphingolipid and IL-17 signaling pathways could serve as a potential strategy for targeted therapy in WD liver fibrosis. Figure 7C integrates the key active components, target genes, and signaling pathways identified in this study, illustrating the synergistic mechanism of BSHXHZF. The active components of BSHXHZF interact with inflammatory cytokines and the key enzymes in sphingolipid metabolism, thereby blocking Th17 cell differentiation and HSC activation. This finding highlights the core feature of BSHXHZF, which is its ability to simultaneously target the inflammatory microenvironment and sphingolipid metabolic dysregulation through multi-component synergy, thereby providing a mechanistic basis for its multi-target therapeutic effects against Wilson's disease liver fibrosis.

This study is limited by the following aspects, First, although copper metabolism disorder is a core pathological feature of Wilson's disease, dynamic changes in hepatic or serum copper ions were not systematically detected, making it impossible to clarify the regulatory effect of BSHXHZF on copper homeostasis and its associative mechanism with improved liver fibrosis. Second, although DELncRNAs were screened through analysis and their expression changes were validated, the specific molecular mechanisms through which they regulate liver fibrosis remain to be analyzed in depth. In addition, we found that the IL-17 and sphingolipid metabolism pathways possibly mediate the therapeutic effects of BSHXHZF. However, the expression changes and functional relevance of key nodal molecules in these pathways were not validated at the molecular level. In the future, we plan to further investigate the synergistic mechanism of BSHXHZF's multi-components on the “LncRNA-signaling pathway” regulatory network so as to provide a more comprehensive theoretical basis for integrated traditional Chinese and Western medicine treatment of WD liver fibrosis.

## Conclusions

The interaction between sphingolipid and IL-17 signaling pathways is crucial for regulating inflammatory responses and the progression of WD liver fibrosis. This potential therapeutic effect broadens the treatment options and provides new hope for patients, especially when existing Western medications have limited efficacy or when patients experience adverse reactions to them. Therefore, the application of TCM is of immense significance. By gaining an in-depth understanding of the interactions between these two signaling pathways, new strategies can be developed for managing WD-induced chronic liver disease, ultimately improving patient outcomes. Such approaches facilitate the translation of basic research into clinical applications and open new avenues for researchers in related fields, promoting communication and collaboration across disciplines.

## Data Availability

The datasets presented in this study can be found in online repositories. The names of the repository/repositories and accession number(s) can be found below: PRJNA1227770 (SRA).
