# Supplementary material for: Mechanistic insights into the therapeutic effects on liver fibrosis in Wilson's disease: a transcriptomic and network pharmacology-based approach
Source: Front Med (Lausanne). 2025 Jun 10;12:1581623. doi: 10.3389/fmed.2025.1581623 (PMC12185468; doi:10.3389/fmed.2025.1581623)
Supplement: Supplementary file 1 [file Table_1.docx]

**Table S1. Primer sequences used in RT-PCR**

| Primer name | Primer sequences (5ʹ→3ʹ) |
| --- | --- |
| *GAPDH* | F: CCTCGTCCCGTAGACAAAATG |
|  | R: TGAGGTCAATGAAGGGGTCGT |
| *NONMMUT060008.2* | F: TGAGTTCAATCCTGGCACCAA |
|  | R: ACAGGGCCAGACATAGAGGT |
| *ENSMUST00000153523* | F: GGTTCCCACCTGTAACCCAG |
|  | R: CCAGAAATGAGCCCACGAGT |
| *NONMMUT096375.1* | F: CCTGTGTGTTGTGTGCATCTC |
|  | R: GACATAGGCACCCGTCACTT |
| *Oprd1* | F: TTTGGCATCGTCCGGTACAC |
|  | R: CAAACGGCCACGTTTCCATC |
| *Ppp2r2b* | F: GTTGGCAGCCCAACATGTAA |
|  | R: GCAAGCCTTGGGCTGAGTT |
| *Sgpp2* | F: GTTCTCTACGCTGGTGTGTCT |
|  | R: GCAGGGTAGGTCAGAGCAAT |
